# Portable microsystem integrates multifunctional dielectrophoresis manipulations and a surface stress biosensor to detect red blood cells for hemolytic anemia

**DOI:** 10.1038/srep33626

**Published:** 2016-09-20

**Authors:** Shengbo Sang, Qiliang Feng, Aoqun Jian, Huiming Li, Jianlong Ji, Qianqian Duan, Wendong Zhang, Tao Wang

**Affiliations:** 1 MicroNano System Research Center, Key Lab of Advanced Transducers and Intelligent Control System of the Ministry of Education &College of Information Engineering, Taiyuan University of Technology, Taiyuan 030024, China; 2 Shanxi Academy of Medical Sciences & Shanxi Dayi Hospital, Taiyuan, 030032 Shanxi, P.R China

## Abstract

Hemolytic anemia intensity has been suggested as a vital factor for the growth of certain clinical complications of sickle cell disease. However, there is no effective and rapid diagnostic method. As a powerful platform for bio-particles testing, biosensors integrated with microfluidics offer great potential for a new generation of portable point of care systems. In this paper, we describe a novel portable microsystem consisting of a multifunctional dielectrophoresis manipulations (MDM) device and a surface stress biosensor to separate and detect red blood cells (RBCs) for diagnosis of hemolytic anemia. The peripheral circuit to power the interdigitated electrode array of the MDM device and the surface stress biosensor test platform were integrated into a portable signal system. The MDM includes a preparing region, a focusing region, and a sorting region. Simulation and experimental results show the RBCs trajectories when they are subjected to the positive DEP force, allowing the successful sorting of living/dead RBCs. Separated RBCs are then transported to the biosensor and the capacitance values resulting from the variation of surface stress were measured. The diagnosis of hemolytic anemia can be realized by detecting RBCs and the portable microsystem provides the assessment to the hemolytic anemia patient.

Lab-on-a-chip (LOC) devices are a promising platform for the preparation, handling, manipulation, and analysis of bio-particles (DNA, virus, bacteria, cell, and small molecules)^1^,^2^,^3^,^4^,^5^ because of small sample size and low power and reaction time requirements, as well as portability and versatility in design^6^. Based on LOC approaches, a multifunctional dielectrophoresis manipulations (MDM) device can be integrated with a peripheral circuit to achieve portable, economic and fast diagnosis.

Recently, there has been increased use of these portable LOC devices for the manipulation and separation of cells^7^. Many methods include dielectrophoresis, electroosmosis, optical interference, acoustic standing waves, splitting laminar flows, mechanical obstacles and restrictions or magnetic force, have been applied for particle manipulation. The use of DEP has the advantage over these more traditional methods because labeling is not required. Youlan *et al*. achieved cell separation using a dielectrophoretic microfluidic device^8^. The ability to capture blood cells was shown by Paul *et al*. using a saw-tooth dielectrophoretic microchannel^9^. A microfluidic device for the continuous manipulation of biological cells with dielectrophoresis was designed by Debanjan *et al*.^10^. Chen *et al*. used a microfluidic chip for the separation of plasma from undiluted human whole blood samples using dielectrophoresis and capillary force^11^. These studies have mainly focused on the separation of different cells from mixtures, but none of these devices offer subsequent analysis or treatment design. Therefore, there is a significant need for the integration of MDM with other miniaturized devices, such as a biosensor and a test circuit of biosensor.

There is increased use of biosensors to provide a wide variety of vital biomedical applications of diagnostics that use cells^12^, DNA^13^, or protein^14^,^15^. As a new micro-scale and label-free technology, surface stress biosensors have immense potential to meet the demand for better sensor quality^16^. Micro-cantilevers and micro-membranes are two sensitive elements of a surface stress biosensor, but micro-cantilevers have lower sensitivity in liquid medium^17^. A micromenbrane-based surface stress biosensor has been studied^14^. In a test system with a surface stress biosensor, wired devices limit the flexibility of the test environment^3^. Integration of a surface stress biosensor with MDM would allow fast and high sensitivity detection of molecules or cells.

For hemolytic anemia caused by the premature destruction of red blood cells (RBCs), there are currently no rapid curative treatments. Hemolytic anemia can result in abnormalities of hemoglobin stability, defects of erythrocyte metabolism, and disorders of erythrocyte hydration^18^. This condition may exhibit an increased level of dead RBCs. The amount of dead RBCs reflects the status of the blood of a hemolytic anemia patient^19^. Therefore, it is possible to diagnose hemolytic anemias based on the content of dead RBCs.

In this work, we designed a novel portable microsystem that consists of an MDM device and a surface stress biosensor test platform to separate and detect RBCs for the diagnosis of hemolytic anemia. The MDM can separate the living/dead RBCs before the high-accuracy biosensor test of separated red cells. Microfabrication and bonding techniques were utilized to fabricate the high precision microfluidic device. A potable signal control circuit composed of a peripheral circuit to power the MDM device and test platform of surface stress biosensor was designed. The diagnosis of hemolytic anemia is achieved by analysis of the obtained electric signal.

## Results

### Manipulation and separation of living/dead cells mixture

**Figure 1:**
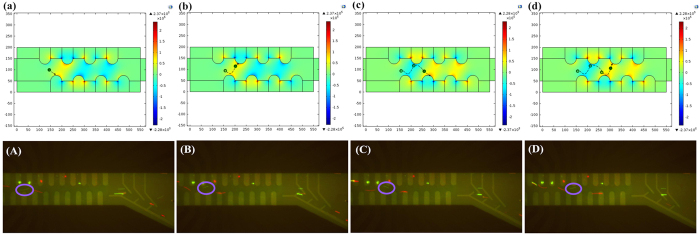
Photograph of living/dead RBCs passing through the microchannel during different phases. (**a**,**b**) The movement of a living RBC in electronic field with simulation (phase = 0°); (**A**,**B**) the movement of a living RBC in electronic field during the experiment (phase = 0°); (**c**,**d**) the movement of a living RBC in electronic field with simulation (phase = 180°); (**C**,**D**) the movement of a living RBC in electronic field during the experiment (phase = 180°).

We first tested the manipulation and separation of a living/dead cells mixture and the simulation and experimental results are shown in Fig. 1. Initially, the cells experience a strong positive DEP (pDEP) force that pushes them into the area of the strong electric field (reddish-yellow parts of Fig. 1a,b). The living cells (green particle in Fig. 1A) experienced strong pDEP from the second down electrode, which is consistent with the simulated results. Then, the cell is moved towards the electrode then arrives at the forth up electrode (Fig. 1B). To monitor the movement of RBCs, the screen captured their elongated shapes, and the statics were rounded. After a reversal of the phase sequence to the electrodes, the distribution of the electric field was switched. The cell is then carried to the next area due to the strong electric field (Fig. 1c,d). During the experiment, the fourth down electrode generated a high gradient electric field, and the living cells (green particle in Fig. 1C) first moved to the up electrodes and then moved towards the down electrode and then were moved to the fifth up electrode (Fig. 1D). Based on this principle, the dead cells (red particle in Fig. 1) experienced the same force during separation and both living and dead cells were pulled rapidly in zig-zag trajectories. The supplementary video (S1) shows the dynamic process.

After particles moved from the focusing region to the sorting region, they can be directed towards one of two exits in either microchannel. Here, the AC signal offers an amplitude of 7 V and frequency of 100 KHz; the Re(*K*_*CM*_) is 0 for living cells and close to 1 for dead cells (Fig. 1). In the structure design of the sorting region, a “T” structure sorting electrode is used to generate a higher gradient electrical field to ensure that the dead cells experience a pDEP and alter their direction of movement towards the bottom channel. The living cells do not experience this force so continue to move forward, as shown in the Supplementary Information video (S1). In this way, the living and dead cells were separated and moved to different collection regions.

### Detection of RBCs

**Figure 2:**
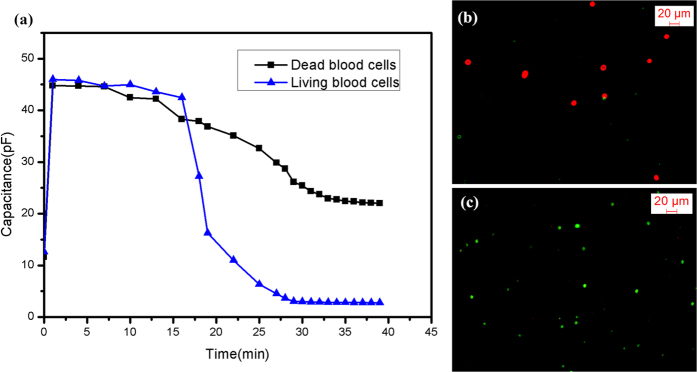
(**a**) The capacitance signals of dead/living blood cells; (**b**) Fluorescent image of dead RBCs; (**c**) Fluorescent image of living RBCs.

The samples collected from the MDM device were moved onto the surface stress biosensor test platform. After that, the living/dead blood cells can be detected according to their capacitance values^20^,^21^. Figure 2 shows the change in capacitance signals during the detection of RBCs: the triangle dotted line represents the variation in the capacitance of living RBCs; the square dotted line shows the trend of the dead RBCs.

At first, all capacitances rapidly increased because the weight of the RBCs suspension liquid is the primary factor affecting the deflection of the membrane. As the solidification of blood suspension liquid occurs, the Van der Waals force and the surface stress induced by the molecular interaction between -COOH groups of the thiol molecules and -OH/=O of the RBCs had a greater effect on membrane deflection. The direction of membrane deflection that was induced by surface stress was opposite to the one induced by the weight of medium^22^. In the final stage, the capacitance decreased and maintained a stable state (the blue line in Fig. 2a). However, the surface stress induced by the dead RBCs decreases due to the destruction of the molecular structure^20^. Additionally, the Van der Waals force and the surface stress induced by molecular interaction decrease, so the capacitance of dead RBCs reached a steady state with a larger capacitance than the living RBCs (the black line in Fig. 2a). The biological experimental results are consistent with the fluorescent images (Fig. 2b,c), and indicate that the potable microsystem can distinguish living/dead RBCs is able to detect RBCs. Based on these results, hemolytic anemia can be diagnosed by monitoring output capacitance.

**Figure 3:**
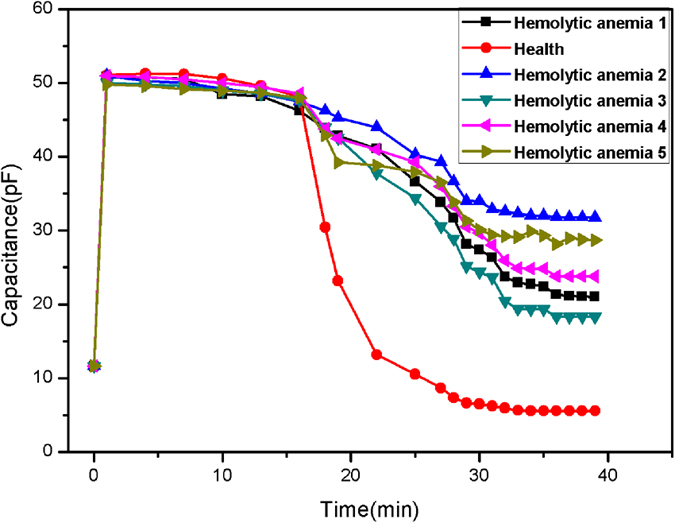
The capacitance signals of healthy blood cells and hemolytic anemia blood cells.

The concentration of dead RBCs in hemolytic anemia patients are significantly higher than the concentration in healthy people. Therefore, diagnosis of hemolytic anemia can be done by detection of dead RBCs in patient’s blood. To verify the technical feasibility of using this potable microsystem, experiments were conducted using the blood from six people: one healthy person, and five patients with hemolytic anemia. All people had O^+^ type blood to reduce potential errors caused by different blood types^23^. Additionally, O type blood is the most predominant of the 4 types so this increased the application value. Figure 3 shows the capacitance signals of the blood samples. It is obvious that the healthy blood cells showed lower final capacitance values (5.90 pF). Additionally, the capacitance signals were different for the five lines (31.24 pF, 28.69 pF, 23.80 pF, 22.08 pF, and 20.01 pF), which indicate different degrees of hemolytic anemia for the patients. Thus, this potable microsystem can be used to diagnose the state of hemolytic anemia by the detection of blood red cells.

## Discussion

Here, a novel portable microsystem was presented that integrates an MDM device and surface stress biosensor to efficiently sort and analyzes RBCs. The MDM device can perform DEP manipulations for guiding, focusing, and sorting RBCs. A unique electrode configuration creates a highly non-uniform electric field for to significantly improve sensitivity. The efficacy of this method was demonstrated using interdigitated electrode arrays to sort living/dead RBCs. The integrated surface stress biosensor can quantitatively detect the sorted RBCs by the variation in the capacitance values. Experimental results reveal that the potable microsystem can not only manipulate and separate living/dead RBCs effectively, but also detect living/dead RBCs, essential for the diagnosis of hemolytic anemia. Future directions of research will include determination of how the capacitance signal can be used to better calibrate the degree of disease.

## Methods

### Principle and Emulation Calculation

DEP is the motion of a particle (charged or uncharged) in a non-uniform electric field and can be used to manipulate target particles in a liquid medium^24^. The direction and magnitude of DEP forces depends upon the conductivity of the particle and the liquid medium. The particles are attracted toward the low electric field regions because the liquid medium is more polarizable than the particle, resulting in negative DEP. If the liquid medium is less polarizable than the particle, the particle will move toward the high electric field regions^25^,^26^, resulting in positive DEP.

The time-averaged DEP force is described by the following equation^27^:


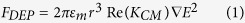


where, *r* is the radius of a spherical particle, *ε*_*m*_ is the permittivity of the liquid medium, Re(*K*_*CM*_) is part of the Clausius-Mossotti (CM) factor *K*_*CM*_, and *E* is the root-mean-square electric field. The Clausius-Mossotti (CM) factor is defined as


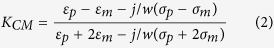


where ε and σ are the permittivity and conductivity, respectively; the subscripts *p* and *m* represent the particle and medium, respectively; *ω* is the angular frequency of the electric field; and *j* is the imaginary unit.

**Figure 4:**
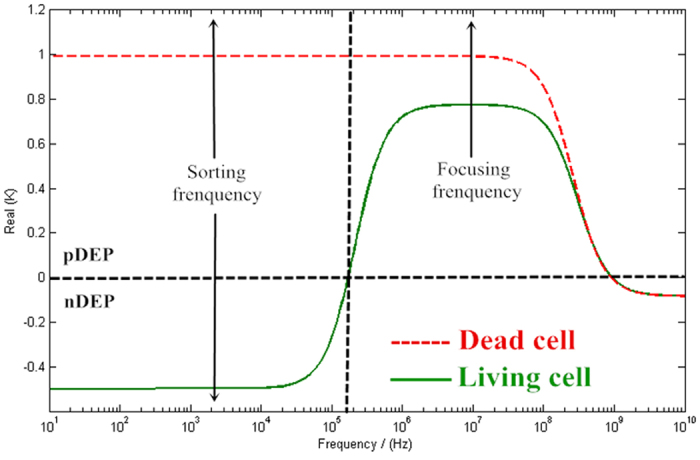
Re(*K*_*CM*_) as a function of electric field frequency for dead and living cells.

The DEP can manipulate and separate living and dead RBCs based on their different dielectric properties. Dead cells have higher permittivity than living cells because of due to their leaky membranes. The high permeability of this leaky membrane allows the diffusion of the components of the suspension medium into the dead cells. Because of this, the cytosol of the dead cell is similar to the suspension medium, causing the conductivity of the dead cell to be lower than that of the living cell. Using equation (2), the relationship between the electric field and frequency can be calculated by MATLAB. As shown in Fig. 4, the real parts of *K*_*CM*_ for living and dead cells are plotted as a function of the frequency. The Re(*K*_*CM*_) of living RBCs range from −0.4 to −0.5 for frequencies from 1 KHz to 100 KHz, representing a negative DEP (nDEP) effect. In contrast, for dead RBC, the Re(*K*_*CM*_) is nearly 1 over the same 1 KHz to 100 MHz frequency range, suggesting a strong positive DEP (pDEP) effect. For 1 MHz to 100 MHz frequency, the Re(*K*_*CM*_) values of both living and dead cells are positive (0.8, 1), indicating a pDEP force. Hence, the signals based on this phenomenon can be used in the MDM device to manipulate RBCs.

The finite element analysis (FEA) software package COMSOL Multiphysics 5.0 was utilized to calculate the trajectories of particles. According to the analysis as shown in Fig. 3, the focusing frequency and sorting frequency were set as 4 MHz and 100 KHz, respectively. The detailed parameters are as follows (Table 1).

**Figure 5:**
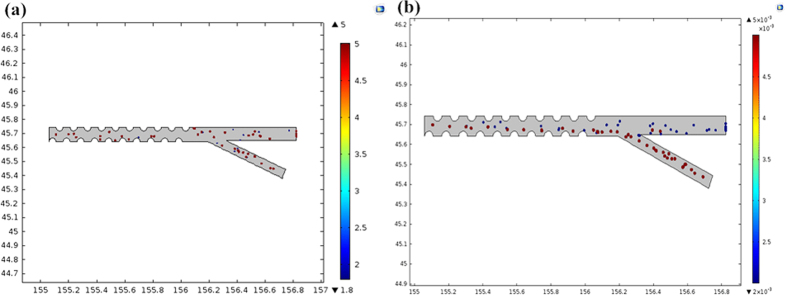
Particle trajectories in the microchannel. (**a**) without DEP force applied; (**b**) with DEP force applied.

This model mainly uses three interfaces: creeping flow to model the fluid flow, electric currents to model the electric field in the microchannel, and particle tracing for fluid flow to compute the trajectories of living and dead RBCs based on drag and DEP forces. Figure 5a depicts the particle trajectories in the absence of the DEP force. The living RBCs are displayed in red and the dead in blue. Figure 5b shows the particle trajectories when they are subjected to the pDEP force. Living and dead RBCs exit the domain through different outlets based on their different particle properties. This simulated model demonstrates the continuous separation of living RBCs from a mixture of RBCs based on dielectrophoresic force. The dynamic changes throughout the procedure are shown in the electronic Supplementary Information (Particle trajectories in the microchannel without DEP force applied (S2) and with DEP force applied (S3)).

### Sample preparation

**Figure 6:**
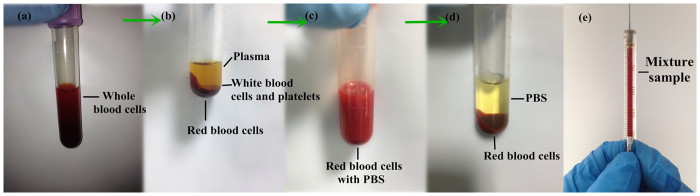
The extraction process of the RBCs from whole blood. (**a**) whole blood cells of donor with hemolytic anemia; (**b**) the blood is separated into three layers after centrifugation; (**c**) RBCs mixed with PBS; (**d**) the mixture separated into two layers after centrifugation; (**e**) haemolytic anemia RBCs mixed with PBS.

Human blood from donors in Shanxi Academy of Medical Sciences was sampled. The protocol was approved by the Institutional Review Board of Shanxi Academy of Medical Sciences. Informed consent was received from all volunteers and the methods were performed in accordance with approved protocol and guidelines. The blood was centrifuged at 3000 rpm for 8 min to collect the cells. The RBCs were extracted and washed with phosphate buffer saline (PBS) three times to remove plasma, buffy coat, and some serum proteins. Mixtures were made of 40 μL of haemolytic anemia RBCs and 160 μL of PBS (Fig. 6).

The mixture cells were labeled with fluorescent markers using the LIVE/DEAD Viability/Cytotoxicity Assay Kit purchased from Life Technologies. This fluorescent kit is a two-color fluorescence cell viability assay based on the simultaneous determination of live and dead cells with two probes that measure recognized parameters of cell viability: intracellular esterase activity and plasma membrane integrity. Live cells are distinguished by the presence of ubiquitous intracellular esterase activity as determined by the enzymatic conversion of the virtually nonfluorescent cell-permeant calcein AM to the intensely fluorescent calcein. The polyanionic dye calcein is well-retained within live cells, producing an intense uniform green fluorescence in live cells. The ethidium homodimer enters cells that have damaged membranes and undergoes a 40-fold enhancement of fluorescence upon binding to nucleic acids, producing a bright red fluorescence in dead cells.

### Design of microfluidic chip

**Figure 7:**
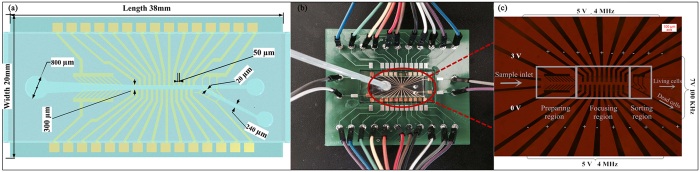
(**a**) Design sketch of the microfluidic chip; (**b**) Photograph of the microfluidic chip and the peripheral control line design; (**c**) The different regions and the electrodes used for application of frequencies and voltages.

The designed microfluidic chip is shown in Fig. 7a. The length and width of the chip is 38 mm and 20 mm, respectively. The depth of the microfluidic channel is 50 μm. Drilled holes on the PDMS microfluidic served as the sample inlet and outlets. The width of the main channel and outlet branches is 300 μm and 240 μm, respectively. The fabricated peripheral control circuit board of the MDM is shown in Fig. 7b and includes a preparing region, a focusing region, and a sorting region, as depicted in Fig. 7c. The preparing region aims to make the particles enter into the channel in an orderly manner. Particles are manipulated in the focusing region, which consists of an interdigitated manipulation electrods array with 50 μm widths and spacing lining on the bottom surface. The focusing region is used to create an alternating electric field. The cell separation occurs in the sorting region, where a “T” style structural sorting electrode aims to generate higher electric field gradients.

### Fabrication of microfluidic chip

The fabrication of the microfluidic is shown in the Supplementary Information (S4). This device consists of the microfluidic channel and bottom micro-electrodes. The silicon wafer was coated by 50 μm-thick SU-8 2050 layer and patterned microfluidic channel with a photomask. Then, PolyDiMethylSilooxane (PDMS) was mixed with primer (10:1 ratio) and was poured on to the silicon wafer with a SU-8 microchannel. After 50 min curing at 90 °C, the solidified PDMS microfluidic channel was peeled off from the silicon wafer. The bottom micro-electrodes were manufactured on a glass wafer using lithographic techniques and sputtering and the electrodes consist of layers of 20 nm Cr and 200 nm gold. Finally, the PDMS microfluidic and bottom micro-electrodes chips were treated with oxygen plasma, positioned under the microscope, and then covalently bonded.

### Peripheral Control

The control signals of the peripheral circuit are provided by the high frequency signal generator. To meet the experimental requirements, the signal generator was designed based on the AD9854, which maintains high-accuracy and fast-reaction. The sorting (100 KHz) and maximum (20 MHz) frequencies can be provided by the dual channels high frequency signal generator, as shown in Supplementary Information S5. The test results indicate that the high frequency signal is sufficiently stable. Two dual channel signal generators provide four AC signals (0~8 V, 0~20 MHz) for the three regions: one for the preparing region (3 V), two for the focusing region (5 V, 4 MHz), and the last for the sorting region (7 V, 100 KHZ). The peripheral control circuit, peripheral control circuit board, and microfluidic constitute the MDM device.

### Biosensor test platform

The biosensor test platform consists of the surface stress biosensor and electronic test platform^22^, as shown in the Supplementary Information (S6). The biosensor chip has two parallel available micro-membranes. HS-(CH_2_)_15_-COOH (16-MHA, 10 mmol/L) was selected as the functional molecules. The thiol molecules can bind strongly to the gold in the top electrode layer of the biosensor chip^20^. The reactions between the micro-membrane functionalized with 16-MHA and analytes will induce a surface stress change, which can cause the membrane to deflect and be detected by the capacitive signal. This tiny change allows the surface stress biosensor to detect the analytes sensitively. The relevant electronic test platform consists of a data acquisition circuit, a digital control circuit, and a data transmission circuit^22^, as depicted in Supplementary Information (S6). It can convert the capacitance signal into a digital signal and achieve real-time data transmission to PC. The ZigBee technology was also used for wireless information interchange from circuit to PC to attain distant detecting and real-time feedback information. The packaged prototype test platform is shown in the Supplementary Information (S6).

### Experimental setup

**Figure 8:**
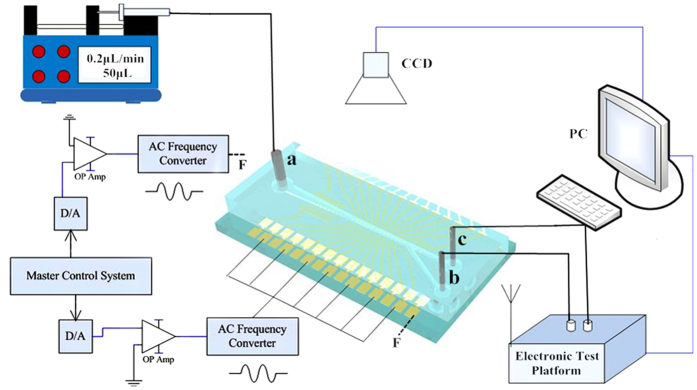
The schematic of the whole test system.

Figure 8 depicts the whole test system integrated with the MDM device and surface stress biosensor test platform. Cells in PBS buffer were extracted by the gas-tight glass syringe (50 μL) and injected into the sample (rate = 0.2 μL/min) inlet “a” through 0.4 mm inner diameter capillary tubing using a pump (11elite 704501, Harvard). Two dual channel signal generators provide four AC signals (0~8 V, 0~20 MHz) for the chip. When blood cells pass through the microfluidic channels, the DEP force will manipulate and separate living/dead RBCs on the focusing and sorting regions of MDM device. A fluorescence microscope (DM3000, Leica) can be used to capture images of the blood cells passing through the microchannel and interdigitated electrodes. Finally, the detached cells are collected from outlet “b” “c”, and detected by the biosensor test platform, respectively.

## Additional Information

**How to cite this article**: Sang, S. *et al*. Portable microsystem integrates multifunctional dielectrophoresis manipulations and a surface stress biosensor to detect red blood cells for hemolytic anemia. *Sci. Rep*. **6**, 33626; doi: 10.1038/srep33626 (2016).

## Supplementary Material

Supplementary Information

Supplementary Movie S1

Supplementary Movie S2

Supplementary Movie S3

## Figures and Tables

**Table 1:** Relevant parameters of the RBCs and suspension.

Category	Parameter	Value
Suspension	conductivity	45 [ms/m]
	relative permittivity	80
Living RBCs	Diameter	5 μm
	conductivity	310 [ms/m]
	relative permittivity	59
	Shell electrical conductivity	0.001 [ms/m]
	Shell relative permittivity	4.4
Dead RBCs	Diameter	4 μm
	conductivity	50 [ms/m]
	relative permittivity	65
	Shell electrical conductivity	0.001 [ms/m]
	Shell relative permittivity	5
