# Supplementary material for: Portable microsystem integrates multifunctional dielectrophoresis manipulations and a surface stress biosensor to detect red blood cells for hemolytic anemia
Source: Sci Rep. 2016 Sep 20;6:33626. doi: 10.1038/srep33626 (PMC5028889; doi:10.1038/srep33626)
Supplement: Supplementary Information [file srep33626-s1.doc]

**Supplementary Information**

**Title: Portable microsystem integrates multifunctional dielectrophoresis manipulations and a surface stress biosensor to detect red blood cells for hemolytic anemia**

Shengbo Sang^1^, Qiliang Feng^1^, Aoqun Jian^1*^, Huiming Li^1^, Jianlong Ji^1^, Qianqian Duan^1^, Wendong Zhang^1^, and Tao Wang^2^

^1^MicroNano System Research Center, Key Lab of Advanced Transducers and Intelligent Control System of the Ministry of Education &College of Information Engineering, Taiyuan University of Technology, Taiyuan 030024, China.

^2^Shanxi Academy of Medical Sciences & Shanxi Dayi Hospital, Taiyuan 030032, Shanxi, P.R China.

^*^Address correspondence to: jianaoqun@tyut.edu.cn, 0086-351-6010029 (Aoqun Jian)

**Supplementary Movies**

Supplementary Movie S1. Manipulation and Separation of RBC. Scanning rate, 0.04s/frame;

Resolution, 768×576 pixels.

Supplementary Movie S2. COMSOL emulation without DEP.

Supplementary Movie S3. COMSOL emulation with DEP.

**Supplementary Figures**

Supplementary Figure S4.

Fabrication process of the microfluidic chip. (a) Spin coating on the silicon wafer; (b) Expose the photoresist; (c) Etch photoresist thin film; (d) Pour PDMS; (e) Peel off; (f) Spin coating on the glass; (g) Expose the photoresist; (h) Etch photoresist and deposit gold; (i) Bond together.

Supplementary Figure S5.

Dual channels high frequency signal generator and its output. (a) Photograph of the signal generator. (b) Sorting frequency determined by the oscilloscope. (c) Maximum frequency determined by the oscilloscope.

Supplementary Figure S6.

Biosensor test platform. (a)The internal structure of biosensor test platform. (b)Testing prototype.

**Supplementary** **Figures**


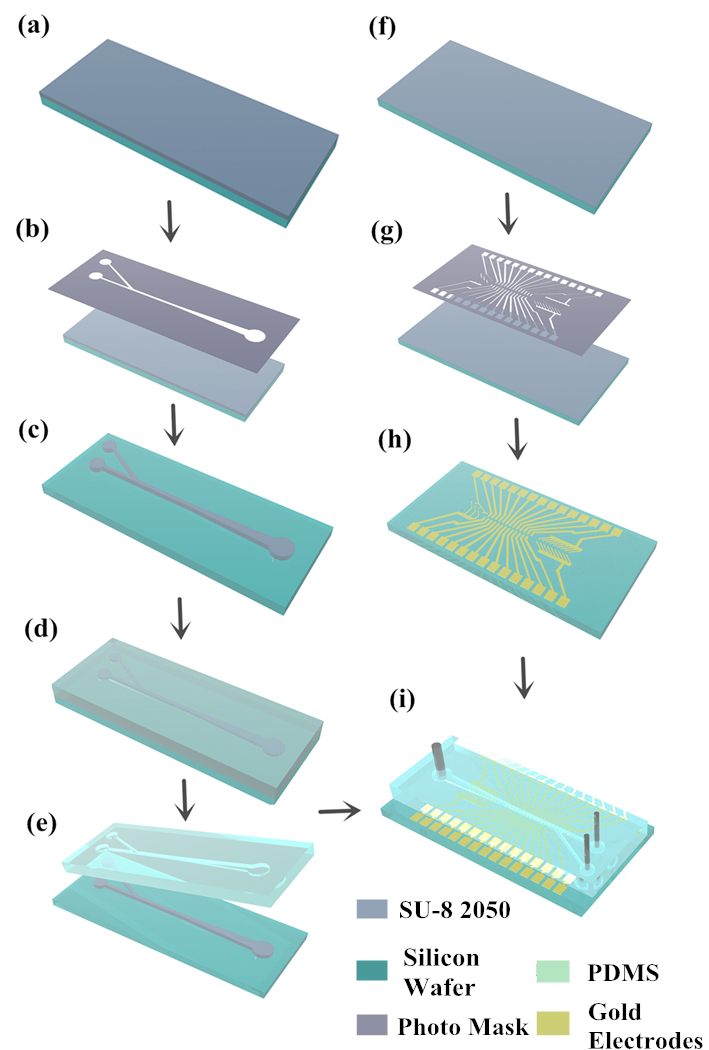


Supplementary Figure S3. Fabrication process of the microfluidic chip. (a) Spin coating on the silicon wafer; (b) Expose the photoresist; (c) Etch photoresist thin film; (d) Pour PDMS; (e) Peel off; (f) Spin coating on the glass; (g) Expose the photoresist; (h) Etch photoresist and deposit gold; (i) Bond together.


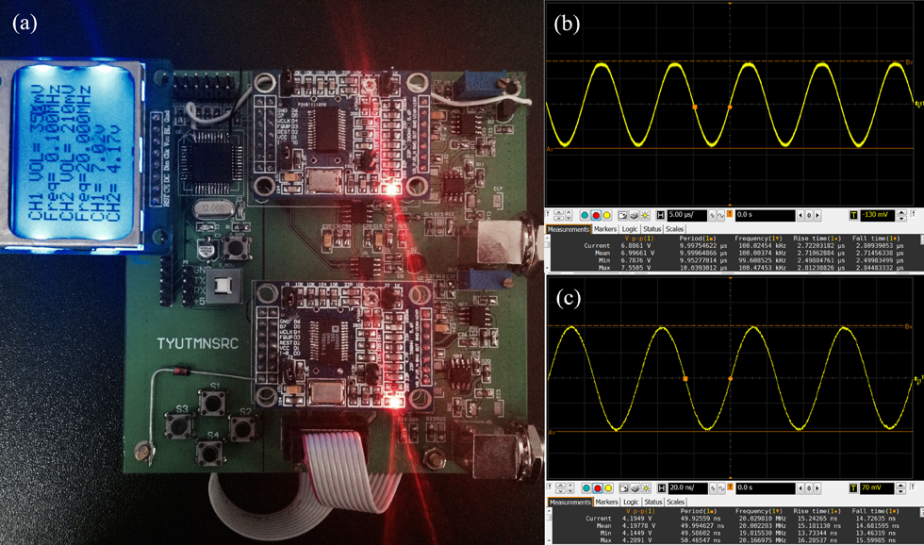


Supplementary Figure S5. Dual channels high frequency signal generator and its output. (a) Photograph of the signal generator. (b) Sorting frequency determined by the oscilloscope. (c) Maximum frequency determined by the oscilloscope.


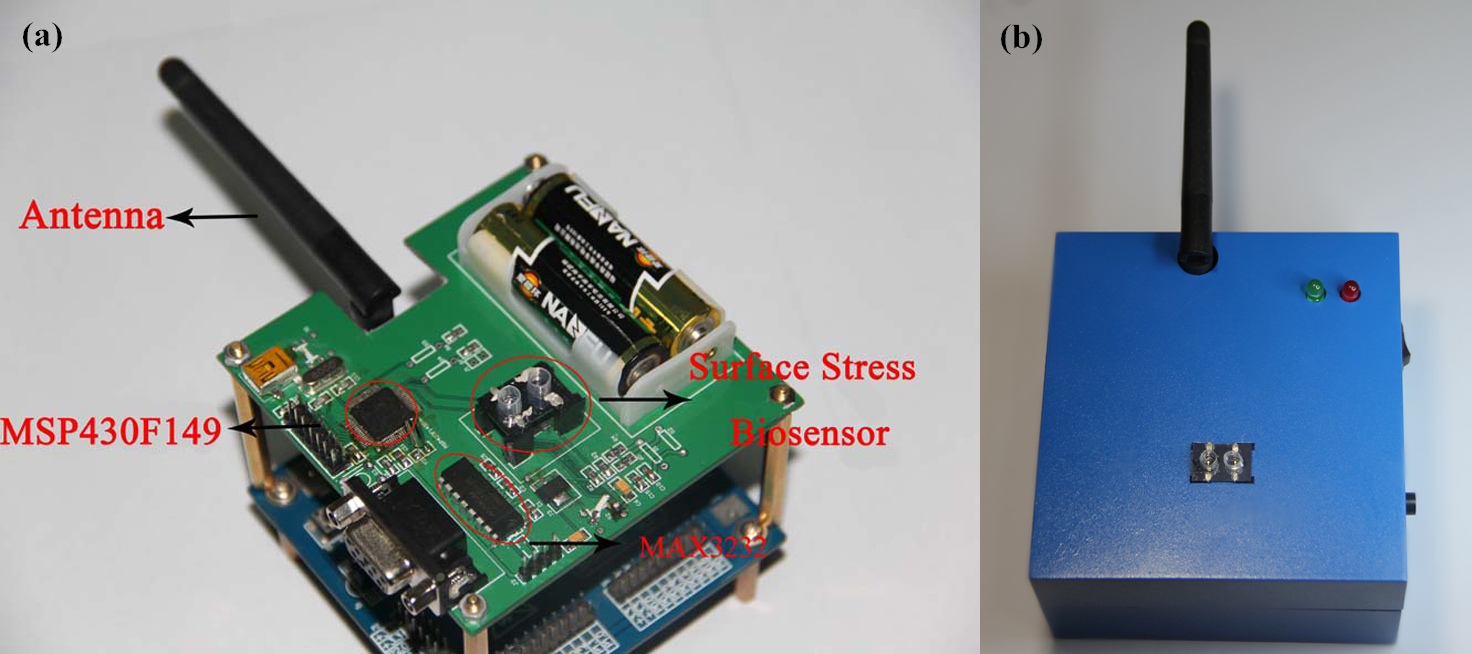


Supplementary Figure S6. Biosensor test platform. (a)The internal structure of biosensor test platform. (b)Testing prototype.
