# Supplementary figures and images for: Portable microsystem integrates multifunctional dielectrophoresis manipulations and a surface stress biosensor to detect red blood cells for hemolytic anemia
Source: Sci Rep. 2016 Sep 20;6:33626. doi: 10.1038/srep33626 (PMC5028889; doi:10.1038/srep33626)

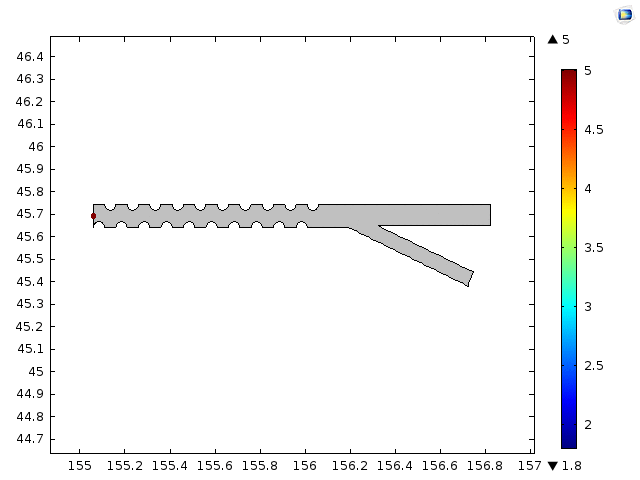

Supplement: Supplementary Movie S2 [file srep33626-s3.gif]

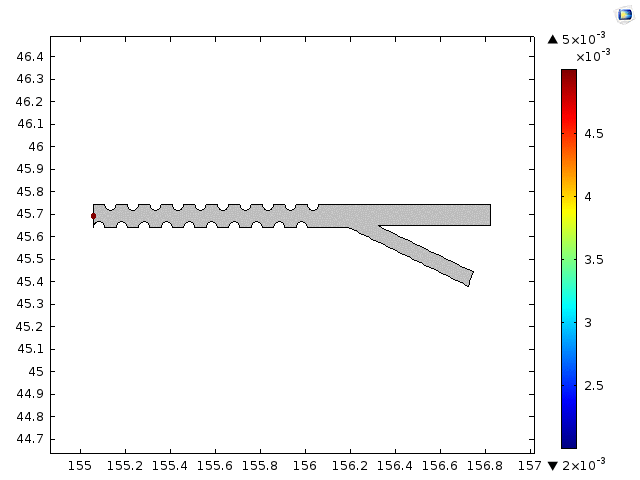

Supplement: Supplementary Movie S3 [file srep33626-s4.gif]
